# The presence of bone marrow cytokeratin-immunoreactive cells does not predict outcome in gastric cancer patients

**DOI:** 10.1038/sj.bjc.6600211

**Published:** 2002-04-08

**Authors:** G de Manzoni, G Pelosi, F Pavanel, A Di Leo, C Pedrazzani, E Durante, C Cordiano, F Pasini

**Affiliations:** Istituto di Semeiotica Chirurgica, Università di Verona, Verona, Italy; Cattedra di Oncologia Medica, Università di Verona, Verona, Italy; Divisione di Anatomia Patologica e Medicina di Laboratorio, Istituto Europeo di Oncologia, Via G. Ripamonti, 435, 2014 Milano, Italy

**Keywords:** gastric cancer, bone marrow micrometastasis, cytokeratin-positive cells, prognosis

## Abstract

The independent prognostic significance of isolated tumour cells in bone marrow is still a matter of debate. This study evaluated the possible association of bone marrow micrometastases with tumour progression and prognosis in patients affected by gastric cancer. Bone marrow aspirates from both iliac crests were obtained from 114 consecutive patients operated on for gastric cancer. The specimens were stained with monoclonal antibody CAM 5.2 which reacts predominantly with cytokeratin filaments 8 and 19. Among 114 cases analysed, 33 cases (29%) had cytokeratine-positive cells in the bone marrow. There was no significant relationship between the presence of bone marrow micrometastases and site, depth of tumour invasion, lymph node metastases, presence of metastases. Patients with cytokeratine-positive cells had a trend towards a diffuse type histology (*P*=0.06). Among the 88 curatively resected patients, median survivals were 40 months and 36 months for cytokeratine-negative and cytokeratine-positive subsets respectively (*P*=0.9). Recurrence of the disease was observed in 39 cases (44.3%); 11 of 24 (45.8%) in the cytokeratine-positive subset and 28 of 64 (43.7%) in the cytokeratine-negative subset. In conclusion in our experience the presence of cytokeratine-positive cells in the bone marrow of curatively resected gastric cancer patients did not affect outcome and its independent prognostic significance remains to be proven before its official acceptance in the TNM classification.

*British Journal of Cancer* (2002) **86**, 1047–1051. DOI: 10.1038/sj/bjc/6600211 www.bjcancer.com

© 2002 Cancer Research UK

## 

The widely used TNM staging system has a high prognostic power, but it cannot predict the outcome in individual patients. Despite radical excision of the primary tumour, almost half the patients with gastric cancer ultimately will die of recurrence. It is likely that this group of patients is understaged probably because of the presence of occult metastatic disease at the time of initial surgery (Kell *et al*, 2000).

Since epithelial cells are not present in bone marrow under normal conditions, identification of tumour cells in the bone marrow by means of immunocytochemistry has been proposed as a method to detect systemic micrometastatic disease (Lindemann *et al*, 1992; Harbeck *et al*, 1994).

Bone marrow micrometastases have been found in over one-quarter of patients undergoing curative resection of gastrointestinal cancer (O'Sullivan *et al*, 1995) and according to some authors they affected prognosis (Schlimok *et al*, 1991; Jauch *et al*, 1996). However the independent prognostic significance of isolated tumour cells in bone marrow is still a matter of debate and remains to be substantiated (Funke and Schraut, 1998; Hermanek *et al*, 1999).

This prospective study was aimed at evaluating the possible association of bone marrow involvement with clinico-pathological features and prognosis in gastric cancer patients.

## MATERIALS AND METHODS

From October 1996 to June 2000, 114 consecutive patients who were operated on for gastric cancer at the First Department of General Surgery of the University of Verona (Italy) underwent bone-marrow aspiration from both upper iliac crests before removal of the primary tumour. The median age of the patients was 67 years (range 27–87); the ratio of men to women was about 2 to 1 (73 men).

Tumours were staged according to the 1997 pathologic classifications (pTNM) of the International Union Against Cancer (Sobin and Wittekind, 1997) and the histological classification followed the criteria of Lauren.

In 92 cases there was pathological confirmation of curative resection (classified as R0), while residual tumour was present in 22 cases, either microscopic (R1) or macroscopic (R2). None of the R0 patients received postoperative chemotherapy or radiotherapy.

### Follow-up

None of the patients was lost to follow-up which consisted of physical examination, routine blood chemistry, tumour markers, abdominal ultrasound (or computed tomography of the abdomen), chest X-ray at 6 months interval and yearly digestive endoscopy; other investigations were performed at the physician's judgement. Recurrences were classified as haematogeneous and intra-abdominal (peritoneal or locoregional), on the basis of imaging studies or intraoperative and bioptic findings in re-operated cases. Only the first site of relapse was taken into consideration.

The median follow-up for surviving patients was 30 months (range 9–49). The 22 cases with R1/R2 resection and the four patients who died postoperatively were excluded from the survival analysis.

### Examination of bone marrow

The preparation of the samples and the immunocytochemical procedures were performed as previously described with some modifications (Pelosi *et al*, 1999a). Briefly, bone marrow aspirates (4 to 6 ml each) were collected in heparinized syringes, diluted with an equivalent volume of RPMI 1640 and sedimented onto a Ficoll-Hypaque density-gradient. The mononuclear cell layer was washed three times in RPMI 1640 and then resuspended at 1/10^6^ cells ml^−1^. This cell suspension was cytospun (Cytospin 3; Shandon Scientific, Cheshire UK) using Shandon Megafunnel disposable sample chambers. Each slide was composed of a monolayered spot of 2.0×1.4 cm containing 3.5–5.0×10^5^ well-preserved mononuclear cells. Slides were fixed in pure acetone at 4°C for 5 min and stored at −20°C in aluminium foil until using.

Cytospin slides were incubated in a moist chamber with monoclonal antibody CAM 5.2 (IgG_2a_, Bechton Dickinson, CA, USA) which reacts predominantly with cytokeratin filaments 8 and 19. The reaction was developed with a commercially available immunostaining kit (EnVision AP – Dako) according to the manufacturer's instructions.

The specificity of each assay was verified by replacing the primary antibody with unrelated mouse IgG in buffer at comparable dilution. Cytospins from fine needle aspiration biopsy of surgically excised primary gastric adenocarcinomas stained in parallel were employed as external positive controls, and myeloid cells of each bone marrow aspirate were used as internal negative control. Finally, as a control group, bone marrow aspirates were collected from 31 patients with a variety of non-malignant diseases (five sigmoid diverticulitis, six benign haematologic diseases, nine cholelithiasis, 11 abdominal aneurysm). In a set of experiments we used bone marrow aspirates from unrelated, non-malignant diseases artificially contaminated with a known number of gastric cancer cells taken by fine needle aspiration biopsy from surgical specimens to determine the sensitivity of our immunocytochemical method in the detection of cytokeratin-reactive tumour cells as previously reported in detail (Pelosi *et al*, 1999b). These contamination tests showed that cytokeratin-positive cells were detected in expected quantities in all mixtures up to the final dilution of 1/10^6^ bone marrow cells, either sedimenting mononuclear cells or granulocytes (data not shown).

**Figure 1:**
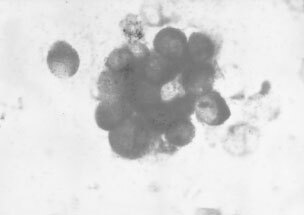
Individual and clustered gastric cancer cells in the bone marrow highlighted by strong labelling for cytokeratin in the cytoplasm and with a cell morphology consistent with that of tumor cells. (APAAP method, 1000 x, oil immersion).

At least four slides of bone marrow aspirate for each patient were evaluated independently and blindly by two observers (F Pasini and G Pelosi) without knowledge of patient's identity or clinical course of the disease. Specimens were considered as being positive if strong labelling of the cell cytoplasm was seen and the cell morphology was consistent with that of epithelial tumour cells (Figure 1

).

### Statistical analysis

The intra- and interobserver reproducibility was evaluated by analysis of variance and Spearman's rank test respectively. Significance of differences between patients with cytokeratine-positive cells and patients with cytokeratine-negative cells was assessed by a chi-square test. Survival curves were computed according to the Kaplan-Meyer method, and compared by the log-rank test. End point was considered death from gastric cancer. Deaths from different causes were considered as censored observations at the time of death.

## RESULTS

Tumour cells in bone marrow were highlighted by a strong cytoplasmic labelling and presented almost exclusively as individual cells (94%); both individual and clustered tumour cells were generally larger than normal mononuclear cells.

Control experiments showed that normal bone marrow elements on cytospin preparations of both clinical material and a control group of non-cancer patients were negative for cytokeratins, with the exception of a few plasma cells and megakaryocytes which were however morphologically identifiable.

Among 114 cases analysed, 33 cases (29%) presented cytokeratine-positive (CK+) cells in the bone marrow. Of the 88 patients evaluable for survival, 24 presented CK+ cells (27.3%). In this subset, the median number of CK+ cells was 7 (range 1–36) per 10^6^ bone marrow mononuclear cells.

Demographics and clinical characteristics of the cohort are shown in Table 1

**Table 1:**
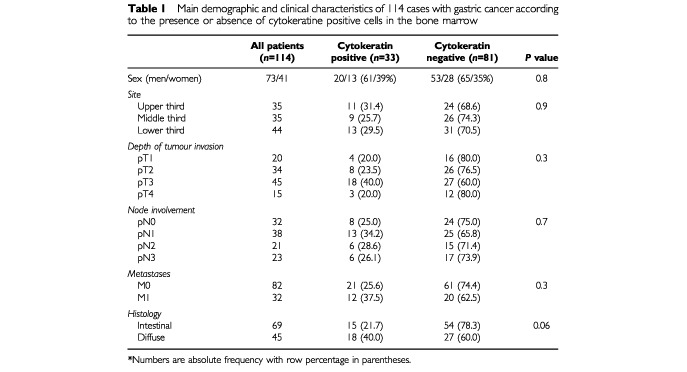
Main demographic and clinical characteristics of 114 cases with gastric cancer according to the presence or absence of cytokeratine positive cells in the bone marrow

. There was no significant relationship between the presence of bone marrow micrometastases and site, depth of tumour invasion, lymph node metastases, presence of metastases. Patients with CK+ cells had a trend towards a diffuse type histology (40% *vs* 21.7% respectively; *P*=0.06).

**Figure 2:**
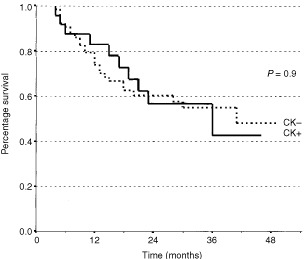
Kaplan–Meier estimates of survival probability in 88 patients affected by gastric cancer who underwent R0 resection, according to the presence (circle line; *n*=24) or absence (square line; *n*=64) of cytokeratine-positive cells in the bone marrow.

Median survivals were 40 months and 36 months for CK- and CK+ subsets respectively (*P*=0.9) (Figure 2

). In the CK+ group there was no correlation between survival and any cut-off value of positive cells.

Among the 88 curatively resected patients, recurrence of the disease was observed in 39 cases (44.3%); 11 of 24 (45.8%) in the CK+ subset and 28 of 64 (43.7%) in the CK- subset. In particular haematogeneous metastases were not associated with the presence of CK positive cells (Table 2

**Table 2:**
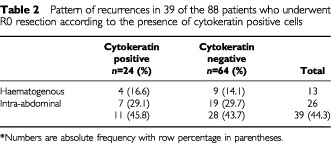
Pattern of recurrences in 39 of the 88 patients who underwent R0 resection according to the presence of cytokeratin positive cells

).

## DISCUSSION

The detection of the presence of epithelial cells in bone marrow has been studied as a prognostic factor in many epithelial malignancies such as breast (Braun *et al*, 2000), small- (Pasini *et al*, 1998; Pelosi *et al*, 1999a) and non small-cell lung cancer (Passlick *et al*, 1999) and oesophageal cancer (Thorban *et al*, 1996). Bone marrow isolated tumour cells are also evident in many patients with gastric cancer, but its prognostic significance is still uncertain (Funke and Schraut, 1998; Hermanek *et al*, 1999). This study was aimed at quantifying the presence of bone marrow involvement and its possible prognostic value in curatively resected gastric cancer patients.

As reported by other studies using iliac crest aspirates the present investigation found that CK+ cells were present in about 30% of the patients with gastric cancer; however percentages of positivity up to 53% have also been reported (Table 3

**Table 3:**
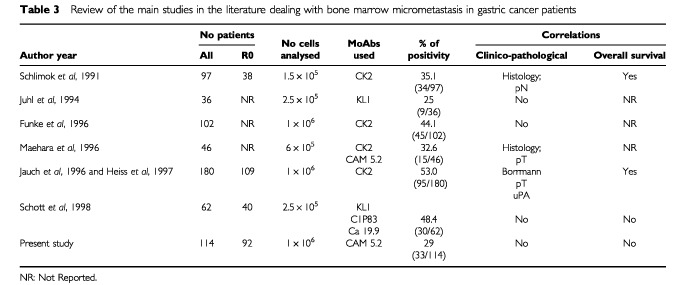
Review of the main studies in the literature dealing with bone marrow micrometastasis in gastric cancer patients

).

The studies addressing the correlation between the presence of CK+ cells and the main clinicopathological factors in gastric cancer have yielded conflicting results. While statistically significant correlations with undifferentiated histology and/or more advanced depth of tumour invasion were found in some studies (Schlimok *et al*, 1991; O'Sullivan *et al*, 1995; Maehara *et al*, 1996; Heiss *et al*, 1997), in others (Funke *et al*, 1996; Jauch *et al*, 1996; Schott *et al*, 1998; Bonavina *et al*, 2001), as well in ours, there was no correlation.

Clinical outcome in gastric cancer has been reported to be affected by the presence of bone marrow infiltration in two studies (Schlimok *et al*, 1991; Jauch *et al*, 1996). While in the report by Schlimok *et al*, 1991 only 38 R0 patients were evaluated, in the recent study by Jauch *et al*, 1996, the survival was analysed in 109 R0 patients. In the latter study, the detection of more than three tumour cells in bone marrow was associated with poorer survival particularly in N0 and T1/T2 subsets. However at multivariate analysis including all conventional risk factors, the presence of CK+ cells was not an independent prognostic factor. In contrast with these results, the present study based on 92 R0 gastric cancer patients, failed to show any association between presence of bone marrow CK+ cells and clinicopathological factors or survival (median survival 36 and 40 months in CK positive and negative subsets respectively; *P*=0.9). Furthermore, no cut-off value was found in relation to survival among positive patients.

The inconsistency among the reports can be explained mainly by two factors: the clinical behaviour of this type of tumour and some problems related to immunocytochemical techniques.

From the clinical point of view, the pattern of recurrence is by large intra-abdominal (peritoneal and locoregional) while systemic metastases are less frequent. Also in our series the pattern of recurrence was mainly peritoneal and locoregional, while haematogenous relapses accounted for only 33%; moreover the percentage of CK+ and CK− cells was similar among the various types of recurrences (Table 2). Theoretically speaking bone marrow contamination can be better associated with systemic spread, rather than with locoregional failure; nevertheless both types of recurrence (intra-abdominal and haematogenous) are probably different steps of a unique and complex process and should therefore be somehow linked. In gastric tumours the overexpression of the protease system, and in particular that of the urokinase-type plasminogen activator (uPA) system, might be associated with the ability of tumour cells to degrade basement membranes, to invade the surrounding matrix and then establish distant metastases. Accordingly, recent studies have shown the correlation of the uPA system either with the presence of CK+ cells in bone marrow (Heiss *et al*, 1997) or with later relapses (Allgayer *et al*, 1997). On the other hand, it should be born in mind that ICC could pick up cells which are non-viable or which are simply in transit (Andreyev and Cunningham, 1997; Kell *et al*, 2000). Therefore correlation between the presence of bone marrow CK+ cells and clinical outcome is not straightforward.

Also the different immunocytochemical procedures used in the various studies might be a source of bias. Different patterns of specificity and sensitivity among the various MoAbs used are known to influence the tumour-cell detection (Borgen *et al*, 1998; Page *et al*, 1999). Due to these problems, also in gastric cancer there was a wide range of positivity (from 25% to 53%).

For these reasons, we paid particular attention to methodological problems. The magnitude of 10^6^ mononuclear cells has been shown to provide a higher probability of detecting even one or two positive cells (Borgen *et al*, 1998; Funke and Schraut, 1998). Another aspect that might be a source of pitfalls is that during the cytospin procedure there is a loss of mononuclear cells and the number of cells rescued on the slides is lower than that counted in the suspension before starting the procedure. We actually counted at least 1×10^6^ mononuclear cells on the cytospin slides. Moreover, we are confident not to have underscored the number of CK+ cells, since in preliminary experiments on artificially contaminated bone marrow specimens, we were able to detect one CK+ tumour cell out of 10^6^ mononuclear cells. In addition, double sedimentation experiments performed on the same samples of artificially contaminated bone marrow aspirates confirmed that all or almost all tumour cells floated in the phase of mononuclear cells (data not shown). Therefore, even in the presence of a minimal bone marrow contamination, it is likely that no tumour cell was missed owing to the different cell layer sedimented.

Another critical point is morphology. As already pointed out, false positive immunocytochemical reactions have been demonstrated between haematopoietic and tumour cells (Borgen *et al*, 1998). Bone marrow tumour cell contamination is still a grey area and it is risky not to take cell morphology into account; experienced observers are therefore necessary for the evaluation of bone marrow cytospins.

The discrepancy in terms of prognostic value of the presence of ITC between the two larger studies calls for a wide co-operation among the researchers in order to establish a consensus laboratory protocol, to verify the neoplastic nature, the biological behaviour and the actual relevance of the isolated tumour cells in gastric cancer.

In conclusion, in our experience the presence of cytokeratine positive cells in the bone marrow of curatively resected gastric cancer patients did not affect outcome and its independent prognostic significance remains to be proven before its official acceptance in the TNM classification.
